# Artificial intelligence behind the scenes: PubMed's Best Match algorithm

**DOI:** 10.5195/jmla.2022.1236

**Published:** 2022-01-01

**Authors:** Lucy Kiester, Clara Turp

**Affiliations:** 1 lucy.kiester@mcgill.ca, Liaison Librarian, McGill University Library, Montreal, Quebec, Canada; 2 clara.turp@mcgill.ca, Discovery Systems Librarian, McGill University Library, Montreal, Quebec, Canada

**Keywords:** PubMed, Best Match, information systems, artificial intelligence, information-seeking behavior

## Abstract

This article focuses on PubMed's Best Match sorting algorithm, presenting a simplified explanation of how it operates and highlighting how artificial intelligence affects search results in ways that are not seen by users. We further discuss user search behaviors and the ethical implications of algorithms, specifically for health care practitioners. PubMed recently began using artificial intelligence to improve the sorting of search results using a Best Match option. In 2020, PubMed deployed this algorithm as the default search method, necessitating serious discussion around the ethics of this and similar algorithms, as users do not always know when an algorithm uses artificial intelligence, what artificial intelligence is, and how it may impact their everyday tasks. These implications resonate strongly in health care, in which the speed and relevancy of search results is crucial but does not negate the importance of a lack of bias in how those search results are selected or presented to the user. As a health care provider will not often venture past the first few results in search of a clinical decision, will Best Match help them find the answers they need more quickly? Or will the algorithm bias their results, leading to the potential suppression of more recent or relevant results?

## INTRODUCTION

Access to information in health domains is an important part of evidence-based practice, with one stage being information seeking. New technologies are increasingly used to improve this access; however, information seekers often lack a basic understanding of the algorithms used to rank records in information resources. The algorithms likely to be encountered in the domain of information professionals are those that are more easily ignored. These algorithms, which affect smaller, everyday tasks, may not be fully recognized because their impact appears smaller than those of more discussed algorithms. For an example, biases involved in facial recognition software algorithms have been highly publicized. By contrast, many librarians, but not members of the general public, may be aware of Google's search algorithm. Taking this one step further, algorithms used within specific databases are even less discussed because they serve smaller domains. However, these algorithms still have an effect on users, especially since they are responsible for the information—and point of view—presented to users.

User search behaviors have evolved in the face of an overabundance of information such that many databases and search engines are adopting machine learning

algorithms to provide more rapid and “relevant” results. Algorithms tend to be highly guarded secrets, giving a competitive advantage, or black boxes that are difficult to demystify. These challenges partly explain why librarians and other information professionals do not— or often cannot—understand exactly how an algorithm ranks search results. This is becoming increasingly problematic as algorithms become more intelligent and prevalent yet also more obscure.

Here, we discuss some ethical implications of artificial intelligence (AI) and highlight ways in which these implications should be reflected in information literacy practices. In particular, we use the example of PubMed, a database that recently updated its search algorithm to use AI. In medicine, the discovery of evidence is a critical part of the clinical decision-making process, which makes PubMed a good example of the necessity of understanding the risks associated with AI. We 1) present background information about user search behaviors and PubMed, 2) introduce several of PubMed's algorithms, with special attention paid to Best Match ranking, and 3) discuss the educational and ethical implications of ranking algorithms that use AI.

As we begin, we define several key concepts as follows:

1. **Algorithms:** Algorithms are defined mathematically as “sets of defined steps structured to process instructions/data to produce an output” [1]. However, an algorithm should not be considered independently from the system in which it functions. In this article, algorithms are considered as systems that interact with other systems, interfaces, and users [2,3].
2. **AI:** AI refers to computer programs that perform tasks requiring intelligence (e.g., problem-solving or learning). AI encompasses machine learning, which is “the science of getting computers to independently learn from—and continuously adapt to—data without being explicitly programmed” [4].
3. **Ethics:** When discussing the ethics of algorithms, we mean that algorithms (including their code, inputs and outputs, and how they are developed and trained) conform to an accepted standard [5]. Within a medical context, we consider the American Medical Association's Code of Ethics, in which “ethical” may be defined as “matters involving moral principles, values, and practices, as well as matters of social policy involving issues of morality in the practice of medicine” [6].
4. **Bias:** Bias is generally considered as deviation from a standard. When discussing algorithmic bias, the focus is on deviations of output, which could remove the algorithm's theoretical neutrality. These deviations are often due to prejudiced assumptions or prejudices in the training data [7,8].

## PUBMED

### What is PubMed?

PubMed is a free database produced by the US National Center for Biotechnology Information for the National Library of Medicine that contains biomedical and life sciences literature [9]. It is one of the most highly accessed free databases, with 3.3 billion individual searches in 2017 and increasing usage each year [10]. It is a vital resource for all evidence-based medicine users and one of the most commonly taught databases by health sciences librarians. PubMed employs Medical Subject Headings (MeSH), a controlled and hierarchically organized vocabulary used for indexing, cataloging, and searching [11]. PubMed is a publicly funded resource, and its usage statistics, search algorithms, and decision-making processes are public record [9].

PubMed's interface has gone through several iterations. In 2013, a relevance sort option was introduced using a classic information retrieval model. Ranking was adjusted “based on how many search terms are found, in which fields they are found, and the frequency of the term across all documents. Additionally, recently published articles are given an artificial boost for sorting” [12]. In 2017, the Best Match algorithm was launched and coexisted with relevance sort so that the National Center for Biotechnology Information could conduct usage testing [12]. In 2020, a new interface and Best Match ranking became the default, and the old PubMed interface and relevance sort were retired.

### User search behaviors in PubMed

General user search behaviors have been strongly influenced in recent years by Google-like systems. Those seeking information want it quickly and easily, especially those seeking health information [13–16]. While many health professionals first reach for a point-of-care tool or application (e.g., UpToDate) that synthesizes preappraised information, many also still search for original research through medical databases such as PubMed [13].

In recent years, with many dramatic changes in the information landscape, information interfaces have had to adapt to remain user friendly. Studies show that most researchers do not go beyond the first page of search results. In 2018, the typical PubMed user was observed to act similarly to those searching the general web, even though PubMed presented its results in a different sort order (i.e., by date), with 80% of clicks happening on the first page [12]. Users are also less likely to search through multiple pages of results if they are using a cell phone [12].

Time constraints and ease of use are two factors that highly impact users of health information in terms of both the tools that are chosen and how the searches are performed [17,18]. This is substantiated by the prevalence of short, overly broad searches, which lead to more results than a user will sift through [4]. Higher numbers of results lead to unsatisfied searchers because there are smaller chances of a user clicking on a document as the total number of documents increase [4]. As librarians, we are fully aware of the difficulties users may have in searching effectively, and changes to databases that improve search results without relying on instruction from experts seems to be a move in a positive direction.

Perceived usefulness and applicability of search results is crucial to users. A recent systematic review identified time and access to knowledge resources as the most common barriers to clinical information seeking and also identified information organization as a frequently mentioned key factor [13]. This need for speed and increased relevance may contribute to PubMed's decision to make Best Match the new default sort mechanism [12].

## PUBMED'S BEST MATCH ALGORITHM

Over time, PubMed has used different algorithms to develop their information retrieval process. Evolving from a date sort to a relevance sort option in 2013, PubMed introduced a ranking based on term frequency-inverse document frequency (TF-IDF), a classic information retrieval model [12]. TF-IDF is used to assign weights to query terms and document index terms, which influence document rankings. In TF-IDF, the weight assigned depends on how often the term is found in one document, which suggests its relevancy, and how often it is found in the overall collection, which suggests its commonality [19]. Therefore, if a query term appears frequently in a small number of documents, the weight for this term will be higher than that for a term found in every document. TF-IDF was the basis for the relevance sort in PubMed, which brought articles with high instances of searched terms to the top of results.

## BM25

PubMed now uses the Best Match 25 (BM25) algorithm, chosen because of its performance [20]. BM25 builds off TF-IDF but calculates document and term frequencies differently. The changes brought by BM25 mostly impact term frequency. BM25 adds two constants (b and k) to adjust assigned weights. The saturation constant (k) is the value that is never exceeded by the term frequency, which reduces the difference between weights of relevant and nonrelevant documents. The constant (b) is used for document-length normalization, which adjusts for document length. In other words, a longer document is not prioritized over a shorter one simply because it has more instances of search terms. These two constants (b and k) can be manipulated to adjust the results of the algorithm [21].

## L2R

The second component of PubMed's Best Match algorithm is learning-to-rank (L2R), which adds a machine learning layer to the search results obtained by BM25. L2R groups multiple different models that have the use of machine learning in common to rank documents. LambdaMART is the specific L2R model used by PubMed. Thus, PubMed's Best Match algorithm has two layers; BM25 is used first to retrieve and rank documents, and then L2R reorders the top 500 sorted results to improve relevancy [12].

In order to help an algorithm learn and improve over time, a data set representing an ideal is used. In machine learning, this data set is considered as a gold standard. If you want an algorithm to learn what is a relevant result for a specific query, you can train the algorithm with ideal query-document pairs (i.e., teaching the program “for this search (query), this is the ideal result (document)”). Since no gold standard query-document pair exists in the PubMed context, PubMed uses a subset of their own logs of users' selection of articles presented on a results page (i.e., click-throughs) [12].

L2R ranks the relevancy of documents by considering different features of the query, the document, and the query-document pair. Example features are publication year, publication type, length of the query, or number of query terms found in a document's title [12]. To determine which features should be included in the algorithm and their weights, PubMed did additional research to identify the features that most improved the performance of relevance sort [12]. However, these features do not carry the same weight in all searches. PubMed's Best Match algorithm uses machine learning to evaluate the value of each feature and adjust its importance in the L2R algorithm.

To summarize, PubMed's new Best Match algorithm first uses BM25 to process the search results and then uses LambdaMART, an L2R algorithm, to rerank the first 500 results. This machine learning algorithm was trained using a combination of document-query features and training data built by user click-through logs.

## Other applications of AI within PubMed

Best Match is not the only feature in PubMed that uses AI algorithms. Query manipulation, author name disambiguation, and automatic indexing of articles are additional examples of how PubMed uses AI to improve search results. Two examples of AI-based query manipulation are query suggestion and query expansion. Query suggestion is when popular queries are presented to the user as they enter terms into the search box, which can result in more successful searches. This feature has been highly used in Google-like systems for quite some time. Query expansion is when the user's query is slightly modified by an algorithm to fix misspelled terms or add controlled vocabulary options. The goal of these modifications is to bring the user query closer to the document collection [4]. This includes Automatic Term Mapping with which many librarians are familiar.

PubMed also uses AI for author name disambiguation, using information such as coauthors and publication dates. In practice, if there are two authors with the name Allen Rickmann, one publishing in 1916 and another in 2005, these two Allen Rickmanns are identified as being less likely to be the same person.

Furthermore, PubMed uses machine learning to index some articles, meaning that a portion of articles are assigned MeSH terms without human intervention via an algorithm with a machine learning component. However, many articles are still indexed manually [22,23]. This automated indexing of articles may have implications for the reliability of MeSH terms in PubMed that are not expanded upon in this article.

## DISCUSSION

### Algorithms as black boxes

Algorithms are much more than mathematical steps. Rather, algorithms are embedded in complex environments consisting of multiple systems and algorithms interacting with each other, which can lead to the existence of a black box. One can understand what was initially put into the black box but receive an output without fully understanding the mechanisms by which it was created. Even if a user understands the mathematical components of a particular algorithm, they might not be able to understand the specific ways in which algorithms interact with each other and manipulate inputs and outputs [24]. As complex computing can be seen as something a bit magical, users can be discouraged in trying to understand the whole environment.

The complexity of an algorithm increases when machine learning is introduced in any part of the system. In adding the element of AI, it becomes more difficult for users to understand what the algorithm does, what decisions it makes, and how it tweaks the initial parameters (e.g., user query) to fit a more common worldview. This creates a challenge to information professionals attempting to teach literacy competencies to information system users. As the complexity of search engine algorithms increases, so too does the level of nuance required in librarian teaching and discussions around such tools.

Furthermore, with machine learning being integrated into algorithmic searching, we see the development of what is known as the accountability gap, which is a gap between what the designer controls, or admits to controlling, and the outputs of the algorithm [24]. This can result in system owners placing the fault of biased or problematic algorithms onto the system itself rather than taking accountability for entering potentially biased information in the first place. This further suggests that designers do not have full control over the system's output, extending this black box effect to the designers themselves, wherein they cannot exactly explain how an output was achieved by the system. So not only do we information professionals have to communicate to users that they may not be able to track the decision-making processes of an algorithm, but we also must highlight the possibility that no one—not even the programmers—may know why results are returned the way they are.

The black box effect of algorithms becomes ethically problematic when algorithms impact users' decision-making. That is, “similar to explicitly persuasive technologies, algorithms can nudge the behaviour of data subjects and human decision-makers by filtering information” [24]. Even if the consequences of biased or filtered information is less obvious or does not seem to have an immediate impact, there are still ethical issues in “nudging the behavior” of system users. Part of the issue is that a system might filter information such that what is presented to a user mostly agrees with a common worldview. As Mittelstadt clearly expresses: “Algorithms inevitably make biased decisions. An algorithm's design and functionality reflects the values of its designer and intended uses, if only to the extent that a particular design is preferred as the best or most efficient option” [24]. Thus, algorithms potentially reinforce societal bias without even presenting the user with another option.

In the case of PubMed's Best Match algorithm, the opacity of the algorithm itself is extended into how the algorithm is trained. The algorithm learns from user click-throughs; however, this is recognized by PubMed as a less-than-ideal gold standard [12]. By relying on user clicks to teach the algorithm, there is a high risk of perpetuating biases on the part of researchers. As only a limited amount of searches are included in the gold standard threshold for educating the algorithm, there is the potential to create a feedback loop where understanding and identifying bias within the algorithm becomes more difficult. When only certain searches meet the standard for educating the algorithm, this means that only certain researchers with their own personal needs and inherent biases are educating the system, thus potentially perpetuating these biases and predilections to affect what all researchers using Best Match see, despite what they may need. As a user, we cannot see what training sets are used, and as a programmer, one only sees the searches and does not know who searched for what or why, which could help understand and identify potential sources of bias, cementing the black box in place.

Another limitation of this standard comes from the assumption that a user clicked on a document because it was relevant. However, users might choose the best of available options or the first mildly relevant document found even if that option does not fulfill their initial information need. Finally, ignoring the clicked document's ranking might introduce bias because users generally click more on the first results [12]. It is increasingly interesting to compare this gold standard to the one used in evidence-based practice.

In evidence-based practice, publication type is taken into consideration when selecting resources to answer a clinical question. For example, a systematic review is ranked higher than a randomized control trial (RCT) due to its level of evidence, as it synthesizes several RCTs. This hierarchy of publication types needs to be taken into account when judging relevance. Currency of the article is also crucial to consider, as medical information has a very short half-life. For example, the Cochrane Collaboration, the internationally recognized publisher of systematic reviews, seeks an update every two to five years for drug-focused reviews [25], and, in general, medical information has a life cycle of about seven years [26]. PubMed identified the importance of publication date and type in the Best Match ranking; however, it is unclear how these features are taken into account or their importance (i.e., weight) in the final ranking [12]. In recent testing of Best Match, keywords appear to be prioritized over date so that in some searches, articles well over ten years old come up in the top ten results. According to evidence-based practice standards, this is too old to be of use. Although Best Match considers publication date and type, it is unclear to what extent it weights them when presenting ordered results.

In general, changes to the PubMed interface and the switch to Best Match were made to respond to users' desires for increased relevancy and speed in addition to usability. However, *apparent* relevance (i.e., exactly matching keywords searched) is not the same as *actual* clinical relevance. For practitioners, a more current systematic review that disproves an RCT should be rated above an older RCT that uses the exact phrase a user searched.

### Transparency

Transparency is achieved through accessibility and comprehensibility [24]. Sharing an algorithm's code makes it accessible, but if it is not comprehensible to most users, do the system owners achieve transparency? PubMed is publicly funded and therefore makes all the information about their system available, thus achieving accessibility. PubMed also works toward comprehensibility by publishing explanations of their algorithms in a language that is understandable to a broader audience. However, considering the complexity of the algorithms, users might not understand how this algorithm actually affects their search results, bringing into question if transparency has been achieved. Furthermore, determining whether AI is used by a system can be nearly impossible for a user. Unless someone has the curiosity to dig into how the Best Match algorithm functions, they could have no idea that an AI algorithm ranks the results to their query and thus be oblivious to the possible implications, both positive and negative.

PubMed's active interface transparency (i.e., what happens when you are actively using the database) is not always equal across every algorithmic action. PubMed sometimes indicates how the user's query has been modified. For example, query suggestion presents different options to users based on their query; when a user starts typing *can,* PubMed suggests different popular searches containing *cancer.* The user can choose to either ignore all suggestions or click on one of them; the user is, therefore, making a conscious choice about their query. However, when reading documentation shared by PubMed, it is not clear how often the system uses other query manipulation (i.e., query expansion) and how obvious the change is to users. For example, when a user types *cancr,* PubMed automatically corrects the spelling and indicates that modification to the user with a message “Did you mean: cancer” above search results about cancer, but not all algorithmic query tweaking is so clearly signposted for users.

An example of this lack of transparency is found within Automatic Term Mapping, which is an example of the query expansion algorithm that maps query terms to MeSH terms or other keywords. PubMed previously showed “search details”—a box that highlighted how a query was mapped by Automatic Term Mapping—on the main results page. With the new PubMed interface, the user cannot immediately see this mapping but has to click into a different page and open a “details” caret to see the mapped terms. The user can still adjust their search in response to this mapping, but the algorithmic actions are less apparent.

Information professionals recognize that users are not always aware of their information needs. Rather, there is often a gap between what the user wants and what the user thinks they want. The reference interview, a discussion between an information professional (e.g., a librarian) and a searcher, is used to help the user better understand their information needs. The problem becomes evident when a user is not aware of the manipulation to their query to adjust it to something that is better represented by the literature or more common within queries. Databases are increasingly moving toward Google-like systems to match current user information behaviors. However, are such systems increasingly becoming black boxes with less transparency?

Even when transparency is achieved when manipulating a user query, there could be ethical implications to these algorithmic manipulations. For example, Google's autocomplete tends to reinforce bias and cultural stereotypes by pointing people away from queries and searches that are unpopular or further from a mainstream worldview [3]. Is introducing query suggestions and query expansions in PubMed reproducing issues of bias already identified in other common algorithms?

**Figure 1:**
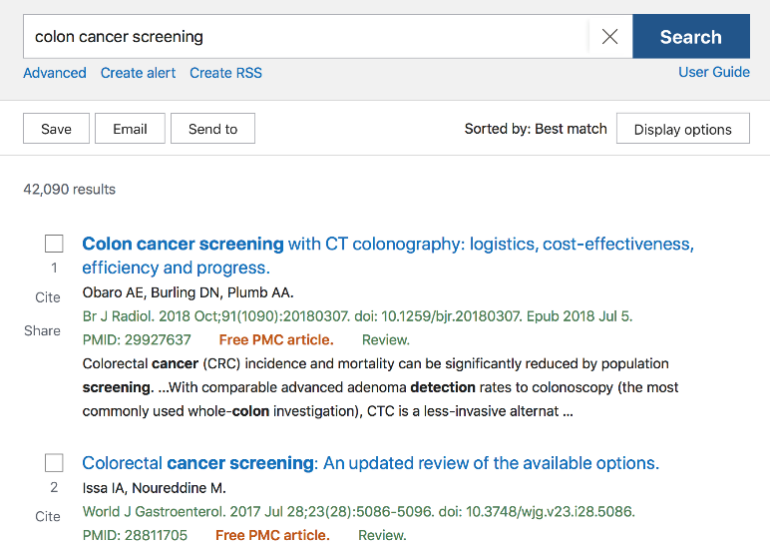
Search results using default Best Match sort in PubMed

**Figure 2:**
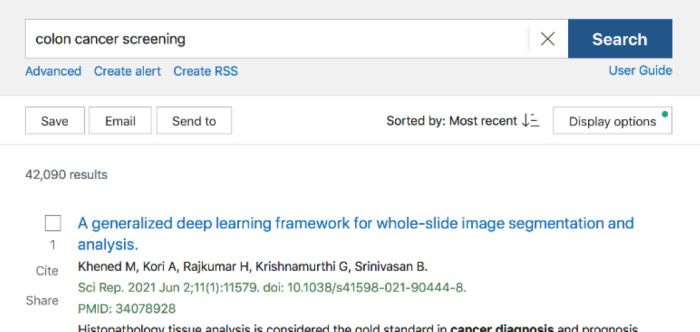
Search results using the Most Recent (by date) sort in PubMed

This leads back to the Best Match algorithm—a choice that is even less transparent than Automatic Term Mapping. When a user is using the Best Match sort order, there is no clear indicator that an algorithm using machine learning is responsible for what articles are presented first. Figure 1 shows the default search results for a simple search on “colon cancer treatment” using Best Match, and Figure 2 shows the search results ordered for Most Recent. Aside from the change in article date at the top (from 2018 to 2021), there is no banner or indicator that PubMed has used AI to reorder items in Best Match.

As Best Match is the default sort order in the new PubMed interface, there is a potential lack of transparency, as users have been trained to not question search order presentations through use of databases like Google. However, in the context of evidence-based practice, currency and publication type are critical. What sort of bias is being introduced in a Best Match sort order? Articles that are clicked more often because they fall into a more accepted world view? Articles that are older, and more cited, but perhaps out of date? For a busy physician with no awareness of the potential ethical biases and concerns of an AI-based algorithm, is the Best Match order safe to use to make clinical decisions?

### AI and the medical community

The medical community is no stranger to the ethics of AI in research and clinical practice. In 2018, the American Medical Association (AMA) passed its first policy recommendations on what they call augmented intelligence [27]. The AMA then developed a full policy on the use of AI intended to educate physicians on how to work with AI algorithms that are positioned to inform clinical care decisions. This policy identifies working with AI as being essential to the future of health care [28]. The policy also recognizes the “thorny challenges” that need to be addressed surrounding machine learning in medical policy, decision-making, and practice.

Critical reception of algorithms is addressed in the AMA's policy; however, the explicit criteria for education and awareness is left abstract, especially the ethics and practices surrounding AI in literature-centered research. PubMed has varied uses—from finding research for systematic reviews to rapidly answering point-of-care-style questions. The fact that searching for literature in a database could involve AI algorithms is not addressed in any policy and may mean that the medical community is not aware of it as a potential ethical problem.

The AMA policy on augmented intelligence highlights several issues (e.g., how a model is evaluated, the importance of focusing on user needs) around the design of health care AI. The AMA also discusses issues regarding transparency that align with the ethical concerns outlined earlier. They highlight the importance of conforming to standards of reproducibility and the need for transparency on the part of designers [28]. PubMed has a certain level of transparency but is perhaps an outlier in this regard. Proprietary databases may not release information on AI algorithms, thus thwarting transparency. However, even in PubMed, reproducibility may be difficult, as rerunning a search after a period of time will result in different result rankings as the Best Match algorithm continues to learn, new articles are published, and click-throughs change. These transparency and reproducibility issues seem to be in conflict with the AMA policy surrounding AI or, if not in conflict, at least adjacent to the need for education surrounding the use of AI.

### Raising awareness through education

We know that users do not go beyond the first page of results, and we know they want increased relevance, whenever, wherever. Is the cost of not using current technology to try to improve relevance too high? Do the possible benefits of improved relevancy—something PubMed argues is reached by the new Best Match algorithm—outweigh the ethical implications of using AI? Even if PubMed teaches its algorithm based on clicks rather than on true relevance (i.e., perceived versus actual relevance), is that better than algorithms that are not built by studying a certain gold standard? Is not the promise of improved relevancy better than a user looking at the first page of results, seeing nothing they perceive as useful, and abandoning the search? We believe in the benefits of using AI and technological advancements to build better search engines, but we argue that they should be evaluated realistically and studied critically.

Perhaps the goal should be to improve education around AI in databases so users are aware of itslimitations, biases, and possible problems. AI has advantages for relevance, especially when considering information behaviors; the value of having relevant information on the first page cannot be understated. PubMed is associated with the National Library of Medicine, a respected and trusted organization. It is also publicly funded, so it achieves above-average transparency. Does that remove the need for education? We would argue absolutely not; even with quality data and a certain level of transparency, education is essential to understand how these systems work. Can we as librarians teach the use of AI with a critical eye? The AMA statement highlights “the promise and limitations of health care AI” [28]. It is important for educators to teach the realities and pitfalls of an algorithm like Best Match, and who is better positioned to highlight both the promise and the limitations of the use of such a tool than librarians?

Librarians should not be stopped by the black box effect of algorithms; rather, they should try to understand how the systems they teach work. The goal of this article is to give tools to librarians to know what questions to ask, to help them understand the potential pitfalls of PubMed's Best Match algorithm, and to feel more confident when looking into any database algorithm. Only through education of the new and growing generation of users—professionals, clinicians, and the public—can we hope to raise awareness and critical understanding around AI, especially AI that would otherwise go unseen or unrecognized. How best to instruct users on choices surrounding algorithms and searching is something that needs investigation. The depth and breadth of educational needs are so varied, and the teaching of AI in databases is a whole new area for librarians to explore; we information professionals are well positioned to become leaders in this educational endeavor.

## NEXT STEPS

Our next steps will be to launch a more practical investigation of how users and the Best Match algorithm interact. We would like to compare search results in Best Match between expert searchers and nonexpert searchers such as students, doctors, and clinical researchers by asking each group to run a search on a query and observing how they search, determine relevance, and select articles to read. Previously, Sampson performed a study in which she used Best Match as a ranker for results from a systematic review search and found that Best Match sort order “placed three times as many relevant records in the top fifty than Most Recent” results [29]. However, her study used searches designed by expert searchers. We wish to test the efficacy of nonexpert searchers by reproducing her research with nonexpert queries.

We are also interested in further investigating features of the Best Match algorithm. We wish to focus on specific elements such as publication dates and material formats to understand how these come into play. Furthermore, we hope to dig deeper into the gold standard used by PubMed to educate the algorithm and find out which types of searchers are included in the standard. As who is using PubMed affects the future of the algorithm and the rankings, we want to know who uses PubMed to potentially uncover biases.

## CONCLUSION

In this article, we provide an introduction to how PubMed uses AI in its Best Match algorithm because it is an open system that documents and shares this information with users. However, even with a certain level of transparency, there are ethical implications to a database using AI. We believe transparency and education are essential to bring awareness to the medical community and information seekers on the role the algorithm plays in their information journey. Instructing users to critically consider how search results are generated and presented, as well as being aware of the level of database transparency, should become a standard part of research training; this will allow clinicians and researchers to take advantage of the strengths of AI while being discerning and ethical in their decision-making process.
